# Supplementary figures and images for: Impairment in facial expression generation in patients with repaired unilateral cleft lip: Effects of the physical properties of facial soft tissues
Source: PLoS One. 2021 Apr 22;16(4):e0249961. doi: 10.1371/journal.pone.0249961 (PMC8061991; doi:10.1371/journal.pone.0249961)

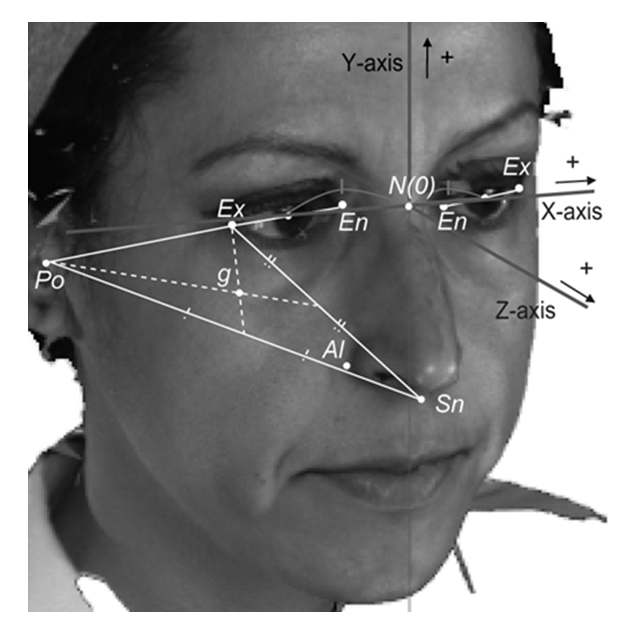

Supplement: S1 Fig — The nasion (N) was defined as the origin (O). The sagittal plane was defined as the plane passing through the origin and perpendicular to the line through the midpoint of the right exocanthion (Ex) and endocanthion (En) and the midpoint of the left Ex and En. The axial plane was defined as the plane passing through the origin and parallel to the line connecting the porion and geometric center (g) of the porion (Po), subnasale (Sn), and Ex on the image projected onto the sagittal reference plane. The coronal plane was defined as a plane passing through the origin and perpendicular to both the axial and sagittal planes. + indicates the positive direction in each axis (Cited from Tanikawa et al. [22] with permission). (TIF) [file pone.0249961.s001.TIF]

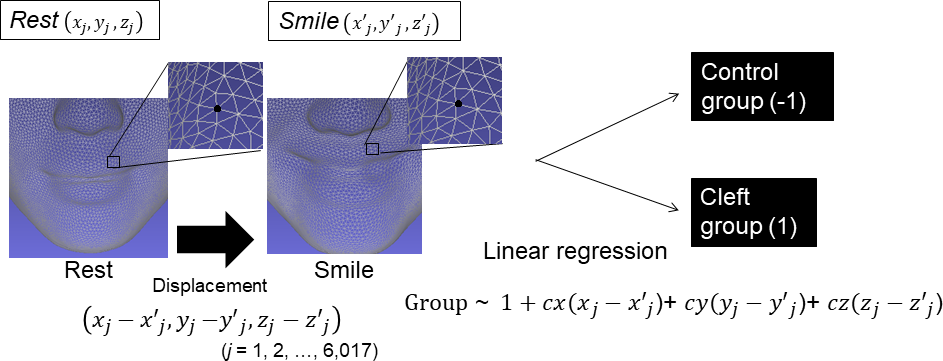

Supplement: S2 Fig — For each semi-landmark of the facial surface, linear regression was applied, where the X-, Y-, Z-displacement values were set as the dependent variables and the Cleft group was assigned a value of 1 and the Control group was assigned a value of -1 as a response variable. The cx, cy, and cz were coefficients for the regression model. (TIF) [file pone.0249961.s002.TIF]

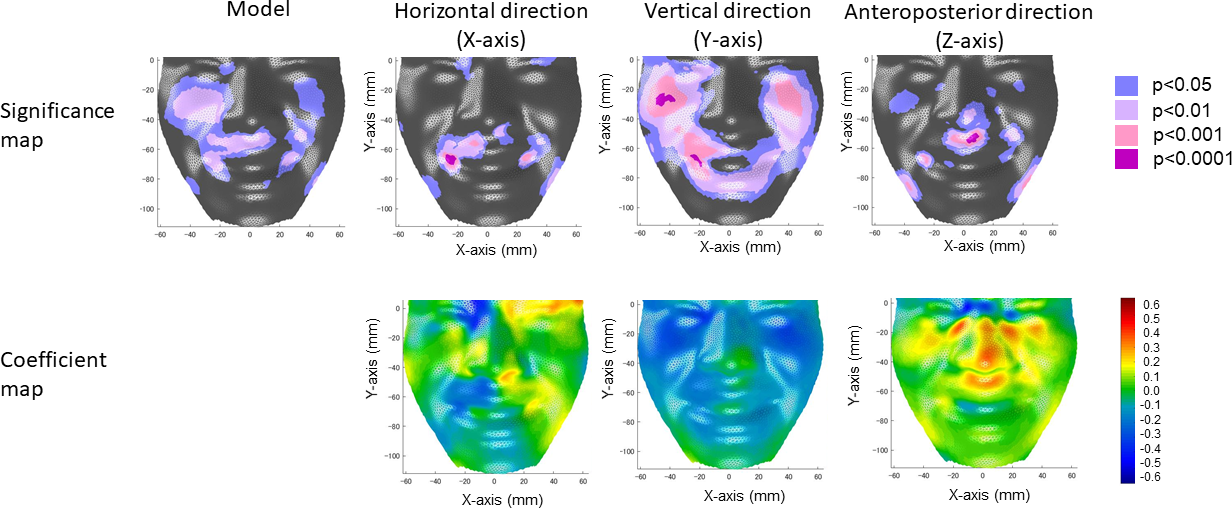

Supplement: S3 Fig — (Model significance [left], horizontal [second left], vertical [second right], and anteroposterior directions [right]). For the significance probability maps, blue indicates P < 0.05; pale pink, P < 0.01; dark pink, P < 0.001; purple, P < 0.0001. Adjustment for multiple comparisons was conducted using the Benjamini-Hochberg method. For the coefficient maps, red and yellow indicate that the coefficient is a positive value, whereas blue indicates that coefficient is a negative value. Displacement was defined as differences in the coordinate values of the face at rest minus those at the peak of smiling. Thus, in the horizontal direction, yellow indicates that the difference in the displacement between the two subject groups (Control group–Cleft group) is a positive value, whereas blue indicates that the difference is a negative value. Displacement was defined as the difference in the coordinate values of the face at rest minus those at the peak of smiling. Thus, in the horizontal direction, on the non-cleft side (right face), yellow indicates a smaller lateral displacement or greater medial movement while smiling in the Cleft group in comparison to the Control group, whereas blue indicates a greater lateral displacement or a smaller medial movement while smiling in comparison to the Control group. On the cleft side (left face), yellow indicates a greater lateral displacement or smaller medial movement while smiling in the Cleft group in comparison to the Control group, whereas blue in the face indicates a smaller lateral displacement or a greater medial movement while smiling in comparison to the Control group. In the vertical direction, blue indicates that a greater downward displacement or a smaller upward displacement while smiling is related to the Cleft group. In the anteroposterior direction in the Cleft group, red and yellow indicates that a smaller retrusive movement or a greater protrusive movement is related to the Cleft group, whereas blue indicates that a g [file pone.0249961.s003.TIF]
