# Supplementary material for: Impairment in facial expression generation in patients with repaired unilateral cleft lip: Effects of the physical properties of facial soft tissues
Source: PLoS One. 2021 Apr 22;16(4):e0249961. doi: 10.1371/journal.pone.0249961 (PMC8061991; doi:10.1371/journal.pone.0249961)
Supplement: S1 Table — (DOCX) [file pone.0249961.s004.docx]

**S1 Table. Mean and standard deviation (SD) of the minimal detectable change for each landmark at the 95% confidence level (MDC_95_) between Session 1 and Session 2**

| **Landmark** | **Elastic modulus**  **(kN/m²)** | **Viscosity coefficient**  **(N･s/m²)** |
| --- | --- | --- |
|  | Mean ± S.D. | Mean ± S.D. |
| **Chk (Left)** | 48.5 ± 24.8 | 111.4 ± 56.8 |
| **Cphs’ (Left)** | 43.2 ± 22.1 | 149.8 ± 76.4 |
| **Cphi (Left)** | 45.1 ± 23.0 | 168.1 ± 85.7 |
| **Ch (Left)** | 20.7 ± 10.6 | 80.0 ± 40.8 |
| **Overall** | 39.4 ± 12.6 | 127.3 ± 39.4 |

*Chk, cheek; Cphs’, crista philtri superior’; Cphi*, *crista philtri inferior; Ch, cheilion*.
