# Supplementary material for: Impairment in facial expression generation in patients with repaired unilateral cleft lip: Effects of the physical properties of facial soft tissues
Source: PLoS One. 2021 Apr 22;16(4):e0249961. doi: 10.1371/journal.pone.0249961 (PMC8061991; doi:10.1371/journal.pone.0249961)
Supplement: S2 Table — (DOCX) [file pone.0249961.s005.docx]

S2 Table. Dental and skeletal parameters of the Cleft group

|  | Cleft  group | Japanese  Norm* | P value |
| --- | --- | --- | --- |
|  | Mean ± SD | Mean ± SD |  |
| SNA (゜) | 74.8 ± 4.5 | 81.5 ± 3.3 | 0.355 |
| SNB (゜) | 75.8 ± 5.4 | 77.8 ± 4.0 | 0.266 |
| ANB (゜) | -1.0 ± 4.0 | 3.2 ± 2.4 | 0.452 |
| SNMP (゜) | 38.4 ± 8.0 | 37.6 ± 6.1 | 0.099 |
| SNPP (゜) | 12.8 ± 6.1 | 8.6 ± 2.2 | 0.071 |
| N-Me (mm) | 130.1 ± 8.3 | 135.7 ± 4.0 | 0.142 |
| Me/PP (mm) | 72.1 ± 5.8 | 74.6 ± 3.0 | 0.292 |
| Ar-Go (mm) | 47.4 ± 6.8 | 53.2 ± 5.7 | 0.142 |
| Ar-Me (mm) | 109.2 ± 8.9 | 115.6 ± 6.8 | 0.066 |
| A-Ptm/PP (mm) | 44.6 ± 4.5 | 51.3 ± 3.8 | 0.329 |
| A-McNamara (mm) | -8.5 ± 5.1 | 2.5 ± 3.1 | 0.332 |
| OJ (mm) | 0.0 ± 4.4 | 3.3 ± 1.0 | 0.482 |
| OB (mm) | 0.6 ± 2.4 | 3.3 ± 1.7 | 0.630 |

* Wada et al. (1977) [28]

A-McNamara, distance from Point A to the McNamara line, defined as a line perpendicular to the FH plane (Or-Po) and passing thorough the N; ANB, A-N-B angle; A-Ptm/PP, point A to Ptm distance projected on the palatal plane (ANS-PNS); Ar-Go, ramus length; Ar-Me, mandibular length; Me/PP, lower anterior facial height; N-Me, total anterior facial height; OB, overbite; OJ, overjet; SD, standard deviation; SNA, S-N-A angle; SNB, S-N-B angle; SNMP, angle formed by the SN plane and mandibular plane (Go-Me); SNPP, angle formed by the SN plane and the palatal plane.
