# Supplementary material for: Impairment in facial expression generation in patients with repaired unilateral cleft lip: Effects of the physical properties of facial soft tissues
Source: PLoS One. 2021 Apr 22;16(4):e0249961. doi: 10.1371/journal.pone.0249961 (PMC8061991; doi:10.1371/journal.pone.0249961)
Supplement: S3 Table — (DOCX) [file pone.0249961.s006.docx]

S3 Table. Dental and skeletal parameters of the Cleft group

|  | Regression statistics | | | ANOVA | | Elasticity(kN/m2) | | | | Viscosity(Pa/s) | | | |
| --- | --- | --- | --- | --- | --- | --- | --- | --- | --- | --- | --- | --- | --- |
|  | R^2 | Adjusted R^2 | MSE | F | p-value | Co-  efficient | MSE | t-stat | p-value | Co-  efficient | MSE | t-stat | p-value |
| *Chk(L)* | 0.39 | 0.24 | 0.88 | 2.58 | 0.004 * | -0.22 | 0.43 | -0.53 | 0.600 | 0.08 | 0.36 | 0.22 | 0.829 |
| *Chk(R)* |  |  |  |  |  | 0.27 | 0.43 | 0.63 | 0.531 | -0.49 | 0.30 | -1.66 | 0.102 |
| *Cphs(L)* |  |  |  |  |  | 0.55 | 0.16 | 3.46 | 0.001 * | -0.08 | 0.19 | -0.43 | 0.670 |
| *Cphs(R)* |  |  |  |  |  | -0.31 | 0.24 | -1.29 | 0.203 | 0.40 | 0.22 | 1.87 | 0.066 |
| *Cphi(L)* |  |  |  |  |  | -0.38 | 0.24 | -1.56 | 0.122 | 0.35 | 0.22 | 1.58 | 0.119 |
| *Cphi(R)* |  |  |  |  |  | -0.18 | 0.26 | -0.68 | 0.500 | 0.08 | 0.22 | 0.36 | 0.718 |
| *Ch(L)* |  |  |  |  |  | 0.44 | 0.33 | 1.31 | 0.195 | -0.49 | 0.29 | -1.71 | 0.093 |
| *Ch(R)* |  |  |  |  |  | 0.26 | 0.31 | 0.85 | 0.401 | 0.05 | 0.28 | 0.18 | 0.857 |

*Chk, cheek; Cphs’, crista philtri superior’; Cphi*, *crista philtri inferior; Ch, cheilion*. L indicate left side; R, right side; R^2, R-squareMSE, mean squared error; F, F-statistics.
