# Supplementary material for: Impairment in facial expression generation in patients with repaired unilateral cleft lip: Effects of the physical properties of facial soft tissues
Source: PLoS One. 2021 Apr 22;16(4):e0249961. doi: 10.1371/journal.pone.0249961 (PMC8061991; doi:10.1371/journal.pone.0249961)
Supplement: S1 Text — (DOCX) [file pone.0249961.s007.docx]

**S1 Text. Intermediate precision of measuring devices for physical properties of facial landmarks**

**Participants**

Eleven adult volunteers (six men and five women, aged 25–32 years) were randomly selected from the Control group for the evaluation of the minimal detectable change in the measurement.

**Data acquisition**

The elastic modulus (kN/m²) and viscosity coefficient (N･s/m²) of facial landmarks including the *cheek* (*Chk*), *crista philtri superior’* (*Cphs’*), *crista philtri inferior* (*Cphi*), and *cheilion* (*Ch*) on the left side were measured using a viscoelasticity measuring instrument (Vesmeter-E100Hs, WaveCyber Corp., Saitama, Japan). The definition of *Cphi* and *Ch* was based on anthropometric investigations described by Mulliken et al. (2001). *Cphs’* was defined as the point on the philtral crest at approximately 12 mm below of the *crista philtri superior* which was defined by Mulliken et al. because of the limitation of measurement from the size of the probe of viscoelasticity measuring instrument. *Chk* was defined as the most prominent point of cheek. The room temperature was set at 25[°C](https://www.rapidtables.com/convert/temperature/celsius.html). Every measurement was conducted three times at each landmark, and an average value of three measurements was employed for statistical analysis. These measurements were repeated on two separate occasions, Session 1 and Session 2, with an interval of 1 week between sessions.

**Statistical analysis**

The minimal detectable change at the 95% confidence level (MDC_95_) was calculated for each landmark based on Bland-Altman analysis [27] using the following equations:

${MDC}_{95}=SEM \times1.96 x\sqrt{2}$ (1)

In equation 1, the standard error of measurements (SEM) was calculated using the following equation:

$SEM = \frac{{SD}_{d}}{\sqrt{2}}$ (2)

The MDC_95_ were calculated for the following regions: *Chk*, *Cphs’*, *Cphi,* and *Ch*.

The MDC_95_ of elastic modulus and viscosity coefficient at each landmark was calculated, and results are shown in S2 Table. The mean value of MDC_95_ in four landmarks was 39.40 kN/m^2^ for elastic modulus and 127.31 N・s/m^2^ for viscosity coefficient, respectively. The maximum value of MDC_95_ was 48.52 kN/m^2^ for the elastic modulus which was found at *Chk* and 168.06 N・s/m^2^ for viscosity coefficient which was found at *Cphi*. The minimum values of MDC_95_ were 20.73 kN/m^2^ and 80.04 N・s/m^2^ , for elastic modulus and viscosity coefficient, respectively, which was found both at *Ch*. The difference in the viscoelasticity between two subjects of each landmark was judged as significant when it is above the MDC_95_ of each landmark.
