# Supplementary material for: Impairment in facial expression generation in patients with repaired unilateral cleft lip: Effects of the physical properties of facial soft tissues
Source: PLoS One. 2021 Apr 22;16(4):e0249961. doi: 10.1371/journal.pone.0249961 (PMC8061991; doi:10.1371/journal.pone.0249961)
Supplement: S2 Text — (DOCX) [file pone.0249961.s008.docx]

**S2 Text. Craniofacial characteristics of the patients in the Cleft group**

A two-sample t-test was used to examine whether there was any significant difference in dental and skeletal parameters between the Cleft group and Japanese normal value [28]. As a result, no significant difference was observed on dental and skeletal parameters between the Cleft group and Japanese normal value (Please see S2 Table).
