# Supplementary material for: Impairment in facial expression generation in patients with repaired unilateral cleft lip: Effects of the physical properties of facial soft tissues
Source: PLoS One. 2021 Apr 22;16(4):e0249961. doi: 10.1371/journal.pone.0249961 (PMC8061991; doi:10.1371/journal.pone.0249961)
Supplement: Attachment — Submitted filename: ResonseToReviewers.docx [file pone.0249961.s009.docx]

**Response to the reviewers:**

**Reviewer #1:**

**General Comments:**

This paper were analyzed facial morphology while smiling in patient with unilateral cleft lip and palate. Correlation between facial displacement and physical property is interesting and should be beneficial information for readers of the journal. However, there is a room for improvement as follows;

**Comment 1)**

Title, Authors started the title with Functional impairment. However, the movement mentioned in this study was just for smiling and at rest position. The movement written in this study technically was not the function. Please reconsider about the title.

**Response 1)**

We thank the reviewer for this important suggestion. Accordingly, we have changed the title to “Impairment in facial expression generation in patients with repaired unilateral cleft lip: Effects of the physical properties of facial soft tissues”.

**Comment 2)**

P9L2; Authors recorded each expression once. Reproducibility of each position was not mentioned even if participants were trained several times. Reproducibility should be added.

**Response 2)**

Thank you for your comment. We have added the intraclass correlation coefficients and 95% confidence intervals for the minimal detectable change of the two facial expressions. (Page 9)

**Comment 3)**

P14L1-2; Expression should be revise.

**Response 3)**

Thank you for your comments. We have deleted, “For details, please see our previous study [19, 22].” Instead, we added a brief explanation (Page 14).

**Comment 4)**

P40L6; Facial expression evaluated in this study was only for the maximum smiling. However, there should be more expressions other than maximum smiling. It would be too hasty to reach the clinical options including surgery from these results. Please reconsider or add the discussion about this point.

**Response 4)**

Thank you for bringing this to our attention. Accordingly, we have added this as a limitation of the present study (Page 41).

**Reviewer #2:**

**General Comments:**

This is a clinical experiment where they test that physical properties of the scar and surrounding facial soft tissue might affect facial displacement while smiling in patients with UCLP. I find the work interesting and the research well performed but I think that needs a better writing.

**Comment 1)**

I am familiar with clinical journals and I know that most of the works are written systematically, but PLoS ONe is not a clinical journal and, therefore, some of the text needs a better explanation. For example, you cannot write "For details, please see our previous study". Instead, you need to explain a summary of the method and cite the study. The manuscript itself should explain all the details of the research to make it repeatable for others. The methods part needs to be more explained.

**Response 1)**

Thank you for your comments. We have deleted, “For details, please see our previous study [19, 22].” Instead, we added a brief explanation (Page 14).

**Comment 2)**

I discovered that to obtain the Elastic and Viscosity parameters you were using a device because it is explained in the legend of the figure. This should be explained in the core text. Moreover, I went to other publication to guess that elastic and viscoelastic parameters were based in the Voigt equation. This should be explained briefly in the text, too. And cite the reference as well. Moreover, the method used to compute the Elastic and Viscosity parameters using the Vesmeter device is defined in one reference, but you should -at least- explain the basics in these documents. Especially because the information you can find in Internet provided by the manufacturer of this device is only in Japanese.

**Response 2)**

Thank you for your comments. We have cited English documents explaining the instrument [11] in the Introduction. We also added a brief explanation about the instrument to the main text (Page 11).

**Comment 3)**

I have a second concern about the results and their statistical treatment. For example, I am not sure that a two-sample t-test can be performed to compare the displacement between two subject groups in each axis. I understand the three coordinates as the same data, so you should use a two-way ANOVA. The same in the physical properties (Elastic and Viscosity parameters) because I understand that they are two parameters of the same Voigt model.

**Response 3)**

Thank you for the comments. To the best of our knowledge, a two-way ANOVA would be inappropriate in this situation because X-, Y-, Z-axis values were related values but has a completely different definition. To clarify this issue, we attached Tables A, B, and C to exemplify data that would be appropriate for a two-way ANOVA data and our data. When compared the data to Table A, showing the data that be applied to a two-way ANOVA, the data in Tables B and C cannot be applied to this analysis because X, Y, Z-displacements cannot be aligned in one column, as each value has a different meaning. Furthermore, when considering weight, height, and chest circumference in the two groups, it is difficult to apply a two-way ANOVA to confirm the interaction between groups and variables. We consider that the weight, height, and chest circumference are similar values to the X-, Y-, Z-values in the present study. As such, we are afraid that the application of a two-way ANOVA would not be appropriate.

However, we assumed that the reviewer was concerned with two possibilities. The first is related to possible associations among the displacement variables in the X-, Y-, Z-axes; the second would be the family-wise type I error rates. Even though we could not confirm the reviewer’s intent, to eliminate any possible concerns, we included two additional statistics. For the latter, we controlled for the false discovery rate using the Benjamini-Hochberg procedure for statistics in facial displacements and facial physical characteristics (Elasticity and Viscosity). For the former, we performed a linear regression analysis to classify two groups when the X-, Y-, Z-values were used as dependent variables and the group was used as an independent variable for each semi-landmark (S2 Fig). This is for considering any associations among the three axis values. The significance of the regression analysis and the significant dependent variable is shown in S3 Fig; the results almost coincided with the previous results, with the exception of the transverse displacement of the ala. We hope that this meets the reviewer's expectations. If the reviewer has any further concerns regarding the details, we are prepared to perform additional analyses or comments.

Table A. Example of data that can be applied in a two-way ANOVA

| No | Weight (g) | Species | Drought. Treatment |
| --- | --- | --- | --- |
| 1 | 737 | PlantA | Control |
| 2 | 818 | PlantA | Control |
| 3 | 716 | PlantA | Drought |
| 4 | 960 | PlantA | Drought |
| 5 | 545 | PlantA | Drought |
| 6 | 445 | PlantB | Control |
| 7 | 443 | PlantB | Control |
| 8 | 529 | PlantB | Drought |
| 9 | 561 | PlantB | Drought |
| 10 | 491 | PlantB | Drought |

Table B. Example of our data. Please note the X, Y, Z-displacements cannot be aligned in one column because each value has different meaning.

| No | X (mm) | Y (mm) | Z (mm) | Group |
| --- | --- | --- | --- | --- |
| 1 | 4.6 | -4.7 | 0.0 | Control |
| 2 | 1.3 | 0.5 | 0.4 | Control |
| 3 | 1.7 | -1.8 | 0.3 | Control |
| 4 | -0.5 | -0.3 | -0.2 | Control |
| 5  … | 1.4 | -1.4 | 0.1 | Control |
| 42 | 0.0 | 7.8 | 0.9 | Cleft |
| 43 | -0.8 | 4.7 | 2.3 | Cleft |
| 44 | 2.0 | 0.4 | 0.7 | Cleft |
| 45 | 0.3 | 1.7 | 2.1 | Cleft |
| 46  … | -0.3 | 0.2 | 0.7 | Cleft |

Table C. Example of our data. Please note Elasticity (kN/m^2^) and Viscosity (Pa/s) cannot be aligned in one column because each value has a different meaning and unit.

| No | Elasticity (kN/m^2^) | Viscosity (Pa/s) | Group |
| --- | --- | --- | --- |
| 1 | 113 | 357 | Control |
| 2 | 152 | 412 | Control |
| 3 | 95 | 359 | Control |
| 4 | 188 | 464 | Control |
| 5  … | 136 | 384 | Control |
| 42 | 386 | 406 | Cleft |
| 43 | 117 | 580 | Cleft |
| 44 | 239 | 373 | Cleft |
| 45 | 214 | 580 | Cleft |
| 46  … | 92 | 373 | Cleft |
